# Possibilities of ICT-supported services in the clinical management of older adults

**DOI:** 10.1007/s40520-016-0711-6

**Published:** 2017-02-11

**Authors:** Miriam Vollenbroek-Hutten, Stephanie Jansen-Kosterink, Monique Tabak, Luca Carlo Feletti, Gianluca Zia, Aurèle N’dja, Hermie Hermens, for the SPRINTT Consortium

**Affiliations:** 1 Roessingh Research and Development, Telemedicine group, Enschede, The Netherlands; 2 Faculty of Electrical Engineering, Mathematics and Computer Science, Telemedicine group, University of Twente, Enschede, The Netherlands; 3 Ziekenhuis Groep Twente, Almelo, The Netherlands; 4 Caretek, Turin, Italy; 5 Sanofi R&D, Chilly Mazarin, France

**Keywords:** Older adults, Chronic diseases, Tele-monitoring, Tele-treatment, Remote monitoring

## Abstract

Services making use of information and communication technology (ICT) are of potential interest to face the challenges of our aging society. Aim of this article is to describe the possible field of application for ICT-supported services in the management of older adults, in particular those with functional impairment. The current status of ICT-supported services is described and examples of how these services can be implemented in everyday practice are given. Upcoming technical solutions and future directions are also addressed. An ICT-supported service is not only the technological tool, but its combination with clinical purposes for which it is used and the way it is implemented in everyday care. Patient’s satisfaction with ICT-supported services is moderate to good. Actual use of patients is higher than those of professionals but very variable. Frequency of use is positively related to clinical outcome. ICT offers a variety of opportunities for the treatment and prevention of frailty and functional decline. Future challenges are related to the intelligence of the systems and making the technologies even more unobtrusive and intuitive.

## Introduction

Demographic aging is a global trend. In the European Union, the number of people aged 65+ will almost double in the next 50 years, from 85 million in 2008 to 151 million in 2060. Among older adults, frailty is highly prevalent and constitutes a major health problem.With increasing age also the chance on having one or more chronic diseases increases. Older adults affected by frailty or having chronic diseases make the most use of community resources, hospitals and long-term care institutions [1, 2]. Most existing health services are symptom-oriented, fragmented and only available at premises of the health care professional or as an inpatient or geriatric health service but there is growing evidence that providing therapy in the home environment is effective [2–5]. Information and communication technology (ICT) services have gained increasing attention due to the development of solutions for tele-monitoring [6] and tele-treatment [7]. Tele-monitoring concerns unobtrusive monitoring of health status, disease-related complaints and/or every day functioning as well as their changes over time. Tele-monitoring makes older adults aware of their health status, its progression and offer possibilities for older adults, their (in)formal carers to undertake actions when needed, and not—as currently often is the case—when escalated. Tele-treatment services support older adults to work on their functional status by exercising independently but remotely supervised by professionals when needed. It is likely that tele-treatment services are more efficient as a single health care professional can treat several people simultaneously and intramural care can be replaced by less costly extramural care. Such services may also be more effective because people can train in their own environment without constraints of available hours of therapists. In addition, tele-monitoring and tele-treatment services put older adults in the driver seat regarding their own health which fits the current trend to put more focus on self-management and patient-centricity. Earlier studies showed that tele-treatment at home is feasible and acceptable to patients and caregivers and can improve their outcomes [8–10]. The Chain of trust (http://www.chainoftrust.eu) shows that both patients and health professionals are willing to benefit from a better access to ICT services. Of course eHealth literacy and user friendliness of telemedicine services need to be addressed properly (http://ec.europa.eu/). Despite this great potential, ICT-supported services are scarcely implemented in daily practice. Reasons for this are among others the fact that clinicians and patients are lost in the variety of services that apparently exist, they doubt about the clinical effectiveness and acceptance of the services and they have no idea on how to start implementation in clinical practice. The aim of this paper is to describe the possibilities of ICT-supported services in the clinical management of older adults and those with chronic diseases in specific. In order to do so, it addresses the current status of ICT-supported services, it presents examples of how ICT-supported services can be implemented in everyday care and it discusses upcoming developments.

## State of the art in ICT supported services

As mentioned, ICT-supported services are scarcely implemented in everyday care. One aspect related to this is the large heterogeneity in the technologies and the clinical purposes for which technology can be introduced. Jansen-Kosterink et al. [11] showed that it is the combination of both, that makes the ICT supported service (Table 1). They used a framework based on Rogante et al. [12] to discern 5 categories of technology: (1) synchronous communication technologies; (2) asynchronous communication technologies; (3) sensor-based technologies; (4) exercise applications to actuate patient to exercise or rehabilitate; and (5) virtual reality and gaming technologies. Concerning the clinical purposes, they roughly discern three purposes:

facilitation (real-time) contact between patients and professionals or professionals mutually,
(safe) monitoring of patients in their daily environment or during exercising,
providing patients the possibility to actually train in their home environment.

They argue that medical professionals can start thinking about the role of technology by reflecting on the way they are currently providing care, and how this can be improved. This will result in concrete choices for technology but also into a new working procedure that is needed when using the technology.

**Table 1:** State of the art of ICT-supported services for physical rehabilitation

Clinical purpose	Clinical examples	Technology category most often used	Example of technology
Services that focus on facilitation contact between patients and professionals	Consultation Information provision	Synchronous communication	Videoconferencing Telephone Telephone + webcam
		Asynchronous communication technology	E-mail Asychronous messaging technology
Services that focus on (safe) monitoring of patients in their daily environment or during exercising	Secure exercising to monitor disease progression Quality/quantity motion	Sensor-based technology	Monitoring biosignals like Electrocardiogram Oxygen saturation Heart rate Blood pressure Motion detection
Services that focus on providing patients the possibility to train in their home environment	Changing behavior in every day life	Synchronous communication	
	Exercising at home	Sensor based technology	Monitoring and feedback on biosignals like Electromyography Activities
		Exercise applications	Web application PC workstation, phone application
		Virtual communities/games	

Another important aspect related to the scarce implementation is the level of scientific evidence available. Regarding the evaluation of ICT-supported services there is much debate. First of all, it is unclear whether clinical effectiveness should be studied at all or as extensive as we currently try. Once we first used a telephone to make our conversations instead of writing letters or visit patients no-one investigated the added value as the intervention we provided stayed exactly the same. Now we are using more advanced ICT but still offer the same treatment. One may also question whether we should investigate the clinical effectiveness. Next to that, there is debate about the study design. As randomized controlled trials (RCTs) are considered to provide valuable evidence about clinical effectiveness, telemedicine research today tries to investigate clinical benefits by adhering to these standards. However, because of the rapidly developing technology and the possibly strong preference of older adults to use or not to use technology, traditional RCTs are difficult if not futile [13, 14]. In addition, as telemedicine services are actually shaped through ongoing interaction with end-users preferably in real life settings, they cannot be recognized as controlled and singular interventions [15]. This argues for new evaluation methodologies [14] like cohort multiple RCTs [16].

However, looking at the state of art, the stage approach of DeChantet al. [17] is a good framework to use. According to this framework, telemedicine assessment preferably follows 4 stages; stage 1 and 2 aim at proving the technical and clinical feasibility of telemedicine interventions. In stage 3 and 4, more global impacts on health care are evaluated like: (1) user satisfaction, (2) clinical effectiveness, and (3) economic benefits. Concerning ICT-supported physical rehabilitation, studies so far almost always include user satisfaction and ease of use for which very positive results are found. Added value in terms of clinical outcome shows that ICT services induce positive health changes and RCTs point out the ICT services are in general as effective as traditional care [11]. Outcomes in terms of cost-effectiveness are hardly there [13, 15] and, when addressed, they merely focuses on reductions in terms of preparation time and travel time.

## Possibilities on how to implement ICT-supported services in everyday care

### Example 1: ICT-supported services in a single hospital or rehabilitation setting

#### The service

**Fig. 1:**
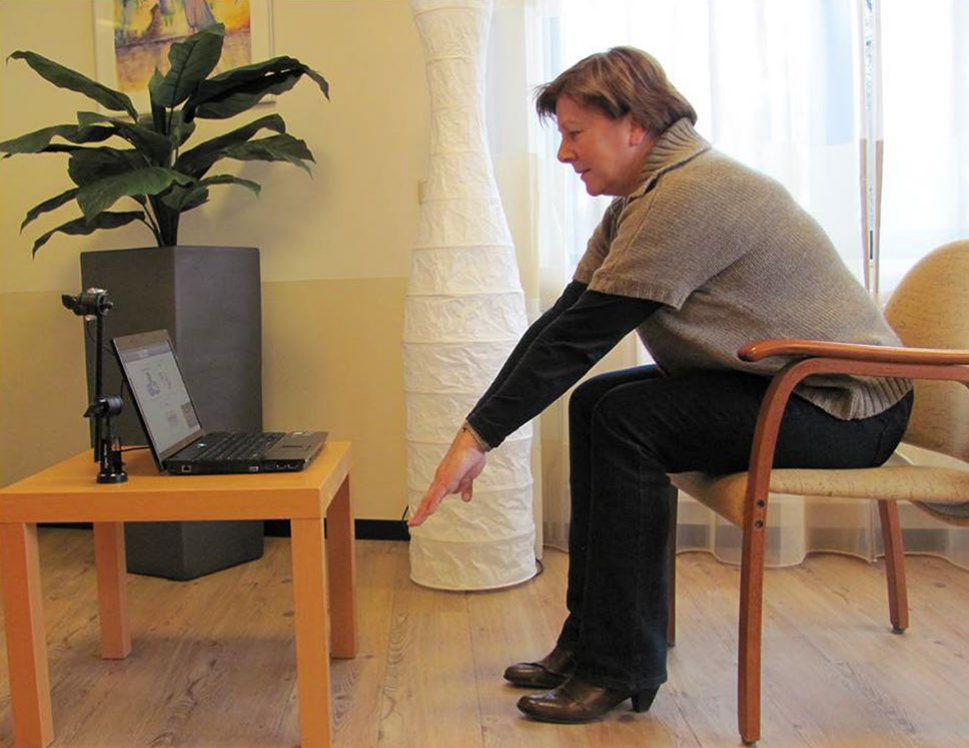
Remote exercise program for physical and cognitive training

This service consists of a notebook with a webcam offering two treatment modules (Fig. 1).

1. A tailored exercise program. A therapist creates an exercise program for each individual patient using exercise videos out of a database for physical training and/or cognitive training.
2. Synchronous contact between patient and physiotherapist using videoconference.

Patients and professionals access the platform by a login and password. Professionals remotely supervise the patient by videoconferencing and using the log files of the system.

#### Implementation in clinical practice

The service was implemented in the Clear project (ICT-PSP 224985) for patients with cognitive problems, stroke survivors, hip or knee osteoarthritis awaiting total joint replacement (TJR), chronic obstructive pulmonary disease (COPD), or chronic pain. Variability in implementation is seen in:

- *Location* Remote exercising can be implemented within a care setting, at home or in the form of a kiosk in the patient’s neighborhood.
- *Timing* It can be implemented before actual treatment to pre-strengthen, after a clinical period as follow-up treatment or during a treatment period as extra-training session of as partial replacement.

In CLEAR, patients with *cognitive problems* were enabled to train independently either at the rehabilitation institute or at home and are monitored by a health professional for about 2–3 months. *Stroke* patients started training at the hospital and continued at home, and at least twice a week at the “kiosk”. The hospital defined the personalized treatment plan and provided the appropriate training material (objects of different shape and size to manipulate, puzzles, printed paths to follow with pens) for home practice. Patients with *TJR* were instructed to train on self-scheduled times at home for 4 weeks on a daily basis pre- and/or post-operatively using a tailored exercise program. Patients with a *whiplash injury* or *severe COPD* (Gold III and V) who visit the rehabilitation center two times a week were given the possibility for extra physical training at home. For patients with *chronic low back pain* (CLBP) and *moderate COPD* (Gold II and III) who visit the rehabilitation center 3 times a week, a treatment day at the center was replaced with the opportunity to train independently at home.

#### Results

For all patient groups the implementation was successfully and a total 673 patients with a mean age between 50 and 79 years, dependent on the diagnosis group, used the remote exercise program (Table 2).

**Table 2:** Satisfaction with of the remote physical and cognitive exercise programs for patients with different chronic diseases

Diagnosis and service implementation	ABI Intramural or extramural remote cognitive training	Dementia intramural remote cognitive training	Stroke Remote physical exercising at home and in kiosk	AO/TJR Remote physical exercising at home pre-or postoperative	COPD Remote physical training as partly replacement	Pain Remote physical training as partly replacement	COPD Remote physical training as addition	Pain Remote physical training as addition
Number of patients (N)	151	48	143	215	36	44	20	16
Age (mean, sd) Gender: % female	Intramural age: 52.6 (9.0) Extramural; age: 56.4 (10.2)	Age: 79.1 (6.6) 54% female	Age: 69.1 (SE 1.0) 45% female	Age: 63.2 (10.9) 63% female		Age : 50(13.2) 43% fernale;		
Ease of use	Low: 13.0% Average: 73.0% High: 14.0%	Low: 2% Average: 58.0 High: 40%	Low: 3% Average: 89% High: 8%	Low: 6% Average: 12% High: 82%		Low: 0.0 % Average: 93% High: 7.0%		
Perceive usefulness	Low: 9.0 % Average: 72.0% High: 19.0%	Low: 0% Average:29% High:71%	Low: 11% Average: 51% High: 38%	Low: 1% Average: 19% High: 80%		Low: 0.0 % Average: 98% High: 1.2%		
Attitude	Low: 7.0% Average: 74.0% High: 19.0%	Low: 0% Average: 25% High: 75%	Low: 1% Average: 96% High: 3%	Low: 2% Average: 10% High: 88%		Low: 13.6% Average: 67% High: 18.5%		

### Acceptance

Acceptance measured according to the Technology Acceptance Model (TAM) [18] showed high scores for ease of use, perceived usefulness and attitude towards remote exercise programs.

### Usage

Usage, considered important as higher training intensity is related to better outcome both in face-to-face rehabilitation and remote training [19–22], was studied for stroke, CLBP and COPD. For stroke the ICT program is significantly more effective than the control group who were residents in the municipalities not served by “kiosks” although there was a large variability between individual patients. Gender (females are more likely to improve), baseline severity of paresis and spasticity as well as adherence to the rehabilitation program are important factors. Actual use appeared to be the strongest predictor; the group which adhered to both the kiosk and the home exercise program showed the largest improvements. However, only 30% of the patients attended the kiosk regularly. This is not related to poor acceptance but to experienced difficulties in attending the kiosks. For those patients with COPD and CLBP were the remote treatments partly replaces face to face treatment 62% (COPD) and 59% (CLBP) showed a clinical relevant improvement. This is as effective compared to a traditional care group (not randomized). Time investment of the professional significantly decreased [23]. Also here, actual use is related to clinical benefit [24].

### Example 2: ICT-supported services in an integrated care setting

#### The service

**Fig. 2:**
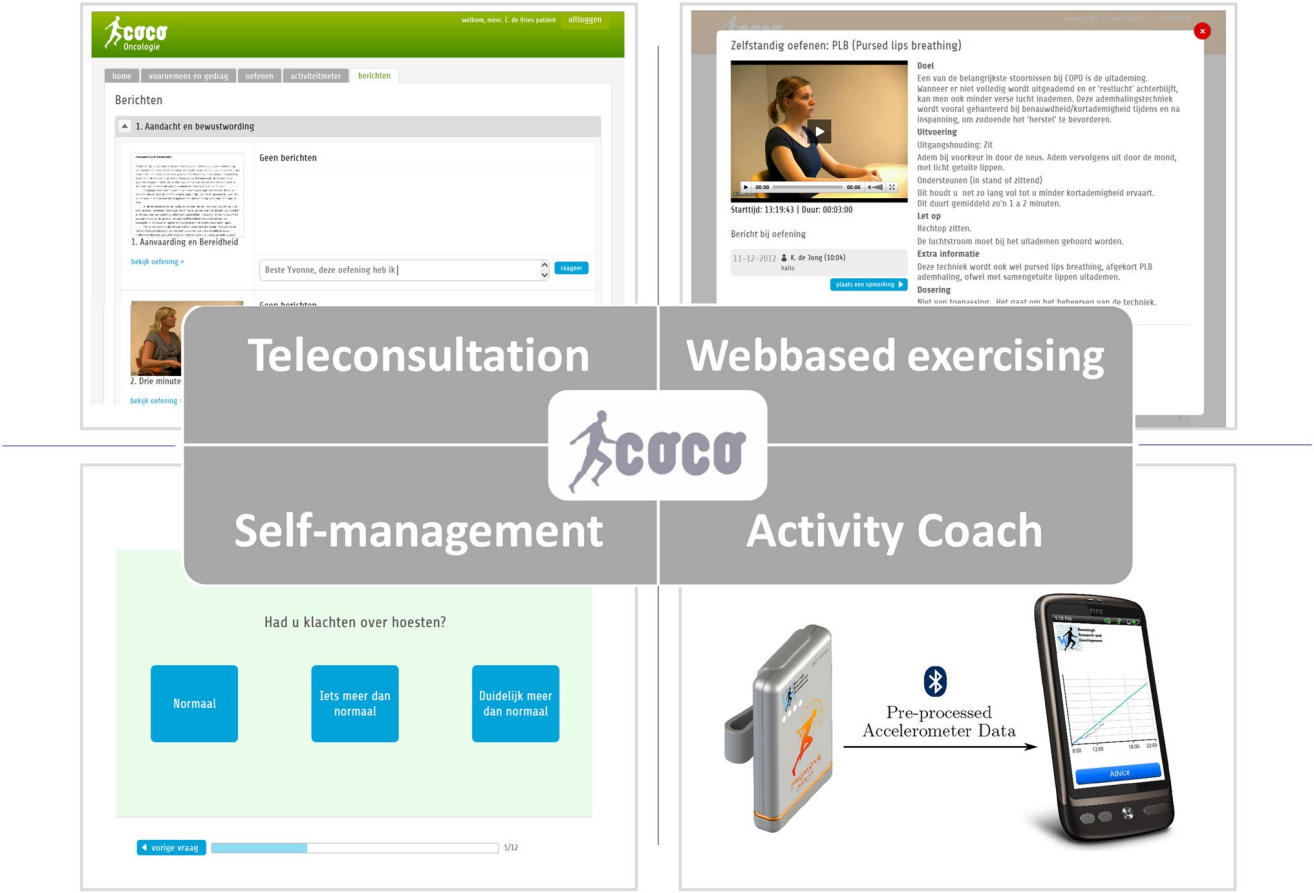
Modules of the ICT-supported services applied in an integrated care setting

A multimodal service platform that enables a multidisciplinary team to offer four different modules (Fig. 2) to their patients:

1. A teleconsultation module for communication between patients and professionals.
2. A web-based exercise program which includes physical exercises for endurance, strength, mobilization, coordination, exercises for activities of daily living like making transfers and psychological exercises. A therapist creates an exercise scheme for every patient. The patient exercises at home and provides feedback to the therapist.
3. An activity coach for ambulant activity registration and real-time feedback. It consists of a 3D-accelerometer (Inertia Technology) and a smartphone. The smartphone shows the measured activity cumulatively in a graph, together with the individually determined cumulative activity the patient should aim for. The patient receives feedback in text messages for awareness and extra motivation.The patient’s measured activity levels were also displayed on the web portal.
4. A self-management module that contains various questionnaires to monitor health status, daily functioning and/or disease-related complaints. For acute hip and arthritis patients, this monitors the level of pain, the influence of pain on daily activities, the level of anxiety and the ability to perform daily activities. For COPD, this consists of a diary for self-treatment of COPD exacerbations with a decision-support system that automatically forms an advice to start medication in case of an impending exacerbation [25].

Through a secure login, both patients and healthcare professionals have access to the modules.

#### Implementation in integrated care pathways

The services were implemented in integrated care paths offered by various healthcare settings (hospitals, nursing homes, primary care physiotherapy practices and a rehabilitation clinic) for 4 different patient groups: acute hip problems, knee/hip arthritis, cancer and COPD. For *acute hip*, during post-operative hospital stay it was used to teach the patient to exercise, make transfers independently. For the follow-up, treatment in primary care of nursing home, it was used to partly replace the treatment at the therapist or to increase the moments of exercising. The therapist determined per patient how and when the different modules were used. For *arthritis*, it was used in the transmural path of the hospital (orthopedic surgeons) and primary care physiotherapist to support patients in dealing with the consequences of their complaints, the development of a more active life style and increasing self-management. Therapist and patient decided together how and which modules to use. For *cancer*, it was used in a standardized 10-week cancer rehabilitation program. One week before treatment and in the 6th week the activity coach module was used. In week 3, patients start using the web-based exercise module next to the treatment at the rehabilitation clinic. From week 11 till week 33, only the web-based exercising at home was used. For *COPD*, it was used in a transmural care path of the hospital and primary care physiotherapists for 9 months. Before start, patients attended self-management sessions, given by a nurse practitioner at the hospital, on how to use the self-management module on a daily basis. The primary care physiotherapist determined per patient the use of activity coach module and selected the exercises in the web-based exercise module.

#### Results

**Fig. 3:**
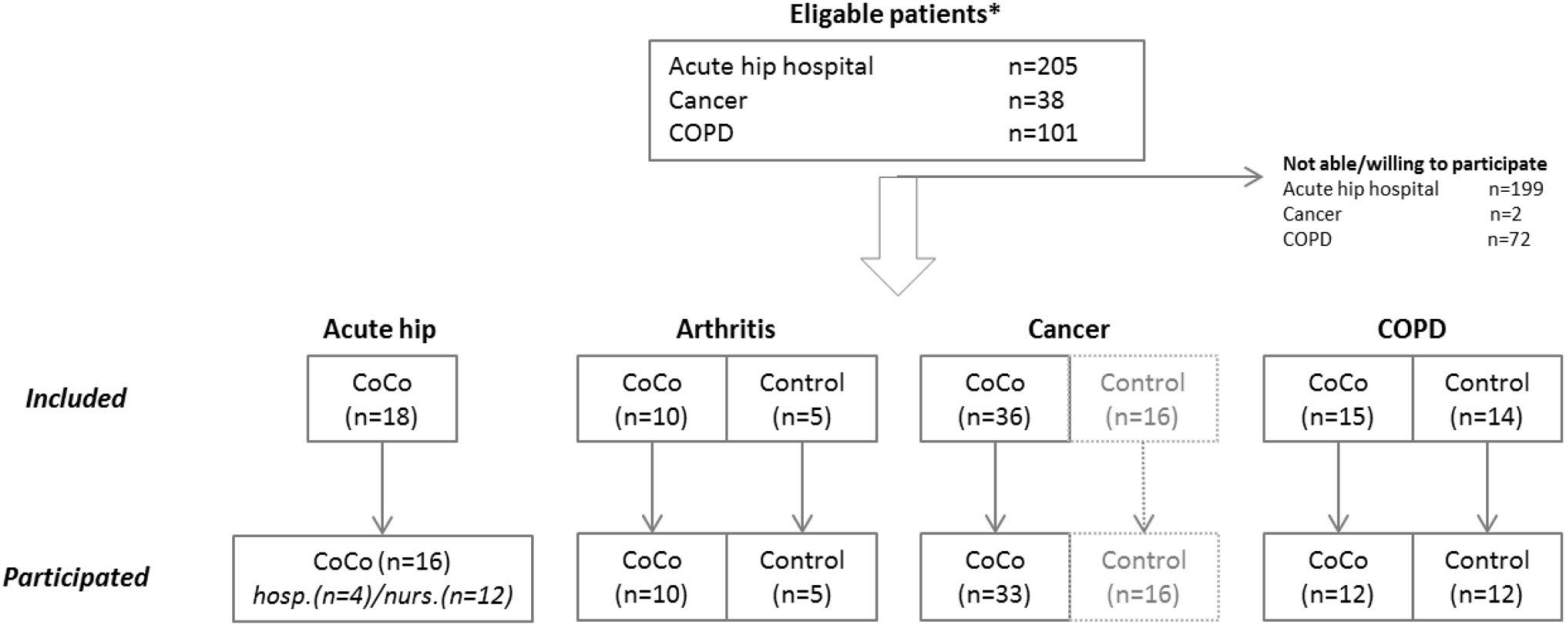
Flowchart for recruitment, inclusion and participation of patients per care path. *Asterisk* eligible patient numbers for acute hip nursing home and arthritis are not available. Acute hip was evaluated by a prognostic cohort study. For the other groups, a controlled trial, randomized in case of arthritis and COPD, and with a historical cohort control group for cancer was performed

In total, 104 patients participated of which 71 were treated using the service modules (Fig. 3). The participation rate was low for both acute hip at the hospital (3%) and COPD (29%). Surgery after a fall appears to be a too big life event resulting in problems like cognitive comorbidities or deliria, making web-based exercising too difficult, but probably also the very high age and lack of ICT experience. However, those hip patients and their professionals who did use the services were very satisfied and together defined a service concept for the nursing home situation where patients are more stable i.e. a “kiosk” where older people exercise together, each having their own personalized exercise scheme. For COPD, the low participation rate was mainly caused by the strict selection criteria with a focus on patients with regular exacerbations and the need for internet access at home. In contrast, the participation rate for cancer was extremely high (95%) which is probably related to the way the telemedicine application is being implemented; a standard program for all patients that start group therapy together.

## Satisfaction

The satisfaction with the received care was assessed through the Client Satisfaction Questionnaire (score: 8–32) and by grading the separate modules (numeric rating from 1 to 10) (Table 3). Overall satisfaction was high with an average score of 27. The acute hip and COPD patients were quite positive about the different modules, whereas the arthritis and cancer group rated the modules less positively. Satisfaction with the web-based exercising modules is the highest and good (≥7), followed by the self-management module and the activity coach, both rated as moderate (in between 5 and 7). The teleconsultation service was not rated as it was hardly used. A high percentage of patients (71%) would recommend using the telemedicine service to others.

**Table 3:** Satisfaction with CoCo services

Care path	Satisfaction with care (CSQ)	Web-based exercising	Self-management	Activity coach	Positive recommend (%)
Acute hip, hospital	At discharge 28.8 (4.4), (*n* = 4)	8.6 (0.6), (*n* = 3)	9 (*n* = 2)	10 (*n* = 1)	100% (*n* = 4)
Acute hip, nursing home	Missing	7.8 (1.7), (*n* = 12)		n/a	92% (*n* = 12)
Arthritis	At discharge 22.4 (4.4), (*n* = 7)	6.3 (1.6), (*n* = 6)	5.1 (1.1), (*n* = 7)	5.4 (2.6), (*n* = 7)	28.6% (*n* = 7)
Cancer	At discharge 28.4 (3.6), (*n* = 16)	6.1 (1.5), (*n* = 17)	6.3 (1.5), (*n* = 17)	5.9 (1.5), (*n* = 17)	44% (*n* = 9)
COPD	At 3 months 26.3(1.3 SE), (*n* = 12)	7.5(1.5), (*n* = 11)	7.9 (1.3), (*n* = 11)	6.8 (2.6), (*n* = 12)	90% (*n* = 10)
Weighted average	27	7.0	6.7	6.2	71%

*CSQ* client satisfaction questionnaire (score 8–32). Data shown as mean (SD) unless stated otherwise. Modules are graded on a scale from 1 to 10

## Usage

Actual use of the services for patients and professionals is expressed as percentage of days they visit the portal during treatment (Table 4). Patients visited the portal 80% of the treatment days. The use by professionals was unexpectedly low: 9–32%.

**Table 4:** Use of the service modules

Care path	Treatment duration (average)	Patients use (average % of treatment days)	Professionals use (average % of treatment days)
Acute hip, hospital	60 days	70%	18%
Arthritis	Missing	Missing	Missing
Cancer	231 days	87%	9%
COPD	256 days	79.8%	32%

ICT supported services as presented are suitable for patients with chronic diseases in various health care setting. In addition, its feasibility is also shown for community dwelling elderly to prevent functional decline and frailty. The PERSSILAA project (http://www.perssilaa.eu) demonstrated the feasibility and acceptability of an online self-screening on frailty and daily functioning as well as a web-based self-management fall prevention program for community dwelling older adults. SPRINTT (IMI-JU-115621) focuses on home services for physical training for community dwelling older adults.The challenge they face here is to make the ICT supported services even more intuitive and unobtrusive. In this respect the project focuses on the sensing part and the user interaction part. Concerning the sensing part it makes use of new sensors and sensor systems integrated in wearables like smart textiles or wristbands that are entering the market every day, that are low cost, easy to don and doff, not interfering with the activities of daily living. In an ancillary study, SPRINTT uses a smart watch (http://www.adamo-vita.it) to detect activity behavior of older adults in their daily environment. The watch communicates with the base station which elaborates, manages and transmits information and requests for assistance to a service center via a mobile network. Also smartphones get increasing sensing abilities enabling quantification of physical and physiological functioning but also social interaction and mental state. In order to use it for coaching and assist clinicians in clinical decision making, it is important to give more meaning to these data by developing decision support systems. An example is built in the Mobiguide project (http://www.mobiguide-project.eu). Concerning user interaction, devices are needed that do not require intensive effort by the user and give personalized coaching taking into account a person’s preference, health and behavioral change states. Within SPRINTT robotic devices are explored to monitor health status, present relevant exercises and motivate the older adult to join the robot with exercising. The next step is the development and implementation of recommender systems to select a set of personalized exercises. These systems initially use expert knowledge to set up a set of rules. By monitoring data, embedded in the system, progress of the individual end user can be monitored and new more personalized advices can be generated.

## Discussion

This paper describes the possibilities of ICT supported services in the management of older patients and those with chronic diseases in specific. To do so, it addresses the current status of ICT-supported services, presents examples and describes upcoming solutions.

Results showed that ICT offers a variety of opportunities. Variety in terms of clinical purposes for which ICT can be used, technological tools that can choosen as well as in the way the services can be implemented into everyday practice. Especially the combination of the clinical purpose and technology makes the ICT-supported service [11]. Once having defined the service it is important to choose the way it is implemented in every day practice in terms of location and timing and whether is it added to or replacing current care. To come to proper decisions a user-centered design process [26] is expected to be useful. In multidisciplinary workshops, researchers, designers and medical professionals can think about the role technology can play in the provision of care starting by reflecting on the way they are currently providing care, and how this can be improved. This can result in concrete choices for technology that can be put into practice but also into a new working procedure that are needed.

Patient satisfaction with ICT-supported services is moderate/good which indicates that patients are willing to adopt telemedicine service. However, in contrast to what theories like the TAM [18] and the Unified Theory of Acceptance and Use of Technology [27] hypothesize, satisfaction is not related to higher use. Results of the cancer group showed on average less satisfaction but high usage. Earlier studies have also shown that there was no significant relation between patient satisfaction and treatment compliance [21]. It is hypothesized that conditional aspects like the possibility to get access to the service or the way the health care professional puts the service into practice are important for actual use. Building in experience sessions with a telemedicine service for a patient might be useful [28] as well as providing the service via a neighborhood facility instead of directly totally independent at home. Cranen et al. [29] showed chronic pain patients prefer this above a home situation.

In contrast to the high use among patients, the actual use among the professionals is disappointingly low. This probably related to the fact that only a part of their patients could take part in the study, thereby causing that the telemedicine did not become part of their regular routine. In addition professional in general have the feeling that introducing these services is something on top of their daily work. However, as professionals are important to help patients understand their disease, the potential benefits of treatment and prevention, and encouraging development of self-management skills [30] their attitude towards telemedicine greatly influences the perception and adherence of the patients. This means that strategies to realize large scale implementation are important. One thing is sure, this requires a behavioral change of health care professionals and urges for proper education.
